# Recurrent Aphthous Stomatitis: Treatment and Management

**DOI:** 10.5826/dpc.1104a99

**Published:** 2021-10-01

**Authors:** Marco Manfredini, Stefania Guida, Matteo Giovani, Nicola Lippolis, Enrico Spinas, Francesca Farnetani, Annunziata Dattola, Eleonora Di Matteo, Giovanni Pellacani, Luca Giannetti

**Affiliations:** 1 Surgical, Medical and Dental Department of Morphological Sciences related to Transplant, Oncology and Regenerative Medicine, University of Modena and Reggio Emilia, Modena, Italy; 2 Department of Surgical Sciences, University of Cagliari, Cagliari, Italy; 3 Dermatology Clinic, Department of Systems Medicine, Tor Vergata University, Rome, Italy; 4 Dermatology Clinic, Department of Clinical Internal, Anesthesiological and Cardiovascular Sciences, Sapienza University of Rome, Rome, Italy; 5 Surgical, Medical and Dental Department of Morphological Sciences related to Transplant, Oncology and Regenerative Medicine, Dental Unit, University of Modena and Reggio Emilia, Modena, Italy

**Keywords:** oral, aphthae, recurrent, autoimmune, treatment, herpetiform, aphthosis, Behçet disease

## Abstract

**Background:**

Recurrent aphthous stomatitis consists of the presence of abrasions or ulcerations located on mucosae (oral or genital).

**Objectives:**

The aim of this article is to review the current literature providing the main causes related to recurrent aphthous stomatitis and insights into treatment and management of this clinical condition

**Methods:**

Articles matching terms that correlated with “recurrent aphthous stomatitis” were searched on PubMed, EMBASE, and Cochrane Library and selected according to their pertinence.

**Results:**

Several forms of aphthous stomatitis have been described, based on the extent (minor, major), morphology (herpetiform) and associations to other signs (Behçet syndrome or more complex inflammatory syndromes). Topical as well as systemic treatments have been described to obtain a faster remission of the aphthosis or to reduce associated symptoms such as pain.

**Conclusions:**

Recurrent aphthous stomatitis can have a mild-to-severe clinical appearance, being mainly localized on the oral mucosa or at the level of the genital area. Different strategies have been described so far for its management and treatment.

## Introduction

Aphtha is defined as a round abrasion or ulceration of oral (or genital) mucosa with a 2- to 5-mm diameter [1]. Lesions are usually covered by a fibrinous pseudomembrane, may be single or multiple, and usually resolve in 10–15 days, but generally recur. Lesions are painful and are often exacerbated by food intake [1]. A specific etiologic factor has not been identified yet.

There are at least 5 forms of aphthosis [1,2]:

1. Recurrent aphthosis minor
2. Recurrent aphthosis ulcer minor (Mikulicz ulcer)
3. Recurrent aphthosis ulcer major (Sutton ulcer)
4. Recurrent herpetiform ulcerations
5. Behçet syndrome

## Methods

A search on the major medical literature databases including PubMed, EMBASE and Cochrane Library was performed. The search was used the following search keys: “aphtha” AND “recurrent” OR “aphtha” AND “treatment.” Each article reporting relevant information on the pathogenesis as well as the management and treatment strategies was considered, and the main findings were included in the current review.

## Diagnosis and Treatment

Recurrent aphthous stomatitis (RAS) [1], is a common oral disease characterized by multiple, small, round or oval mucosal ulcers with circumscribed margins, erythematous haloes, and yellow or gray floors that initially appear during childhood or adolescence. Many diseases [3–10] that affect the oral cavity can have similar clinical presentations, making correct diagnosis difficult and sometimes delaying therapy. Unfortunately, the etiology of RAS of is still unknown.

An RAS diagnosis is based on history and clinical findings [2]. The lesions can range from 1 to several rounded, shallow, painful ulcers and recur in bouts from a few days to few months. There are at least 5 forms of RAS, but the most common are minor (MiRAS), major (MaRAS), and herpetiformis ulcers (HU) [2]. Minor RAS is the most frequent, and it affects about 80% of patients with RAS. Aphthae are small, round or oval, usually with a gray-white pseudomembrane and an erythematous halo [1,2]. It usually occurs on non-keratinized surfaces, particularly the labial and buccal mucosa and the floor of the mouth, although it is uncommon on the gingiva, palate, or the back of the tongue [1,2]. Aphthae usually heal within 10–14 days [2,11,12]. MaRASf is a severe form of RAS that can be observed in about 10% of patients with RAS. Ulcers caused by MaRAS can be larger than 1 cm and often develop on the lips, soft palate and fauces. They persist for up to 6 weeks. MaRAS frequently has its onset after adolescence, is chronic, and lasts for up 20 years or more [12,13]. Herpetiformis ulcers affect about 1%–10% of patients with RAS. Up to 100 ulcers may be present at the same time, and while single lesions measure only few millimeters, they tend to coalesce to become large and irregular. HU are more frequent in women. Despite its name, no association with herpesvirus has been found [12,13].

**Figure 1:**
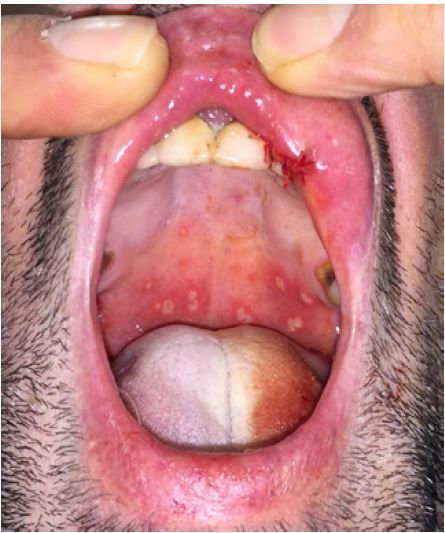
Recurrent aphthous stomatitis in a patient with Adamantiades-Behçet disease.

Behçet disease (Adamantiades-Behçet disease [ABD]) [14,15] is a chronic and multisystemic inflammatory disease, characterized by oral aphthae at its onset, with the successive appearance of ocular, vascular, gastrointestinal, nervous and mucocutaneous lesions (Figure 1). ABD mainly affects young (20- to 40-year-old) males, but no age is spared [14,15]. The male: female ratio is reported to be from 3.1 to 1.1, with the same values being reported in the US and Europe [14,15]. The etiology of ABD is unknown but is most probably caused by an interplay of genetic and environmental factors [14–16].

Multiple oral aphthae and ulcers can be observed in association with several other clinical findings in many autoimmune diseases [3,17] or be induced by medications [18], genetic disease [19,20], and other complex inflammatory syndromes, such as mouth and genital ulcers with inflamed cartilage (MAGIC) syndrome [21], Sweet syndrome [22–28], cyclic neutropenia [29–32], periodic fever with aphthae, pharyngitis and adenitis (sometimes termed PFAPA syndrome) [33–35], nutritional deficiencies [36,37], gluten-sensitive enteropathy (celiac disease), inflammatory bowel disease, and immunodeficiencies [38,39] including HIV infection [40,41].

Recurrent ulcerative lesions of the oropharyngeal mucosa can be associated with many viral or bacterial infections [40,42–44]; therefore, it is important to exclude an infectious etiology before initiating immunosuppressive or an immunomodulatory therapy. AIDS is a progressive viral infection caused by HIV-1/2 viruses, with ulcerative oral manifestations in 8% of the cases [45]. Another cause of ulcerative lesions can be the herpes simplex virus infection (HSV-1/2) when oral and perioral ulcers are frequent symptoms. [43]. Recurrent oropharyngeal ulcers have been described in several infectious diseases as such as: secondary syphilis, tuberculosis, histoplasmosis, Lyme disease, COVID-19, Epstein-Barr virus, and cytomegalovirus infection [44]. The role of *Helicobacter pylori* as an etiology of ulcerative lesions of the oral cavity is still a matter of debate [42].

### Prevention and Therapy

Several therapies, with variable degrees of supporting evidences, are available for the treatment of aphthous stomatitis [11]. Treatment for recurrent aphthous ulcers is aimed at mitigating symptoms, shortening the healing time, and is used as a prophylaxis against recurrence. Most of the treatments are prescribed without studies demonstrating therapeutic efficacy with respect to aphthous stomatitis. Topical regimens are considered to be the standard treatment in mild cases of RAS [2,11,46]. In more compromised cases, topical treatments are likewise very helpful in inducing lesion recovery, but they are often ineffective at prolonging disease-free intervals.

### Medical Care

Topical therapies can include the following: corticosteroids, cyclosporine, retinoids, antimicrobials, anesthetics [47]. Topical corticosteroids are the first-line treatment, and they are used to reduce the local inflammation that induces ulceration. They include dexamethasone (0.5 mg/5mL), triamcinolone (.0,1% gel), fluocinonide (0.05% gel) and clobetasol (0.05% gel). Clobetasol is a class 1 superpotent steroid, and is showing better results [2,46,47].

Immunomodulatory agents, include cyclosporine and retinoids. Cyclosporine has been prescribed as a systemic agent and a topical paste with variable reported efficacy, but it is now frequently used effectively as an oral rinse. Isotretinoin (0.1% gel) and tretinoin in an adhesive base (0.1%), and retinoic acid in an oral base (.05%) have been prescribed for the management of RAS [2,47,48]. Also, systemic isotretinoin has been reported to be an effective therapy for recurrent aphthosis [49,50]. Antimicrobials, including tetracycline, chlorhexidine gluconate, and diluted hydrogen peroxide have shown to reduce the duration and pain of oral aphthae [2,13].

Anesthetics such as topical lidocaine (2% viscous solution, gel, or spray) or benzocaine have been used to reduce the pain associated to RAS [13].

Occlusive and bioadherent agents, such as Gelclair, sucralfate, bismuth subsalicylate, and 2-octyl cyanoacrylate have been successfully used in RAS management, because they can generate a protective coating that shields exposed and overstimulated nerve endings [11].

### Systemic Agents

Systemic therapy includes the following drugs: colchicine, pentoxifylline, steroids, dapsone, thalidomide, pidotimod [16,51,52]. Colchicine was reported to reduce the amount and duration of aphthae in up to 63% of patients with RAS. Treatment over 6 weeks, followed by long-term (years) therapy (1–2 mg daily) is recommended [53]. Combination therapy with colchicine and pentoxifylline, benzathine penicillin, immunosuppressants, or interferon-alfa is possible [52].

Systemic corticosteroids are prescribed as rescue therapy in patients with acute flares and in those who inadequately respond to therapy with colchicine and pentoxifylline. Oral prednisolone or its equivalents, at 10–30 mg daily for up to 1 month can be prescribed during a RAS exacerbation. Intravenous pulse therapy at 100 mg daily for 3 days results in quick improvement for severe cases of RAS [2].

Dapsone (100 mg daily) can be prescribed for oral and genital aphthae; however, rapid relapses can occur after discontinuation of therapy.

Thalidomide at standard (100–300 mg daily) or low (50 mg daily) dosing levels was shown to be effective within 7–10 weeks following administration [54].

In a recent study a new therapeutic high dosage of pidotimod in children with PFAPA (including RAS) showed promising results. Pidotimod is an immunomodulatory agent that increases antigen presentation and promotes adaptive Th1-mediated immunity [55].

### Low Laser Therapy

Four types of lasers have been described in aphthae treatment: CO2, Nd:YAG, diode, and Ga1AS [56]. The main goal of treatment is to decrease pain, healing time, number, and size of ulcers. The main advantage of laser therapy over other treatments is that it can be used for all the causes of the disease without systemic side effects.

### Adamantiades-Behçet Disease

Systemic glucocorticoids are effective for most manifestations of ABD. They are administered at doses of 50–60 mg of prednisone per day, or the equivalent, with a tapering scheme [14,15]. Therefore, other therapies are often used instead of, or as an adjunct to systemic glucocorticoid therapy, such as cyclosporine, colchicine, cyclophosphamide, dapsone, infliximab, interferon alfa-2a, and thalidomide [14–16,49,52].

When mucocutaneous lesions are the main concern in patients with mild-to-moderate aphthosis, the application of topical steroids may obviate the need for systemic therapy, Mild aphthosis may also benefit from the applications of topical sucralfate, topical tetracycline, or a course of zinc sulfate (100 mg orally twice daily) or azithromycin (500 mg orally 3 times weekly) [51]. Azathioprine also proved to be effective in a randomized clinical trial for the prevention of both oral and genital ulcers. In patients with more severe, recalcitrant mucocutaneous lesions, dapsone (100 mg daily) and thalidomide (100 mg daily) are considered particularly effective.

## Conclusions

Recurrent aphthous stomatitis is a common disorder affecting the oral cavity with 3 main presentations: minor, major or herpetiformis ulcers. An RAS diagnosis is often based on history and clinical findings. Recurrent oral ulcerative lesions are rarely associated with several complex inflammatory syndromes and to viral or bacterial infections. Therefore, it is always important to exclude the presence of a systemic or an infectious disease before starting an immunosuppressive or an immunomodulatory therapy.
